# Policy and programmes for mental health in Kerala

**DOI:** 10.1186/1753-6561-7-S5-O12

**Published:** 2013-08-30

**Authors:** K Praveenlal

**Affiliations:** 1 Kerala University for Health Sciences, Kerala, India

## 

A meta-analysis of 13 epidemiological studies shows a prevalence of 5.8% for mental illnesses. Psychiatric morbidity is associated with urban residence, female gender, 35-44 years age group, low socio economic status and nuclear family type. Mental health care facilities are lacking along with a huge gap in professional manpower availability. The available manpower is top heavy and concentrated in the private sector. The National mental health programme aims to assure minimum mental health care for all by application of mental health knowledge in general health care, community participation, and equitable and balanced distribution of resources and integration of mental health with general health services. The programme has failed so far to achieve its objectives due to the absence of a national mental health policy and a lack of mechanism to incorporate political leadership. There is a definite trend towards shifting from public to private health care across all income groups.

A draft mental health policy for Kerala has been submitted with sections on helping family, health care delivery systems, health care services (public sector), health care services(private sector), voluntary sector and administrative aspects. The special features with regard to Kerala in mental health included higher suicide rates, alcohol use, breakdown of marriage and family, problems of ageing, strain due to unmatched parental aspirations and children’s achievements in studies, high rates of migration and single parent families. The seekers of alternate systems are not negligible but the percentage drops in subsequent episodes. Major faith healing centres and initiatives have started hospitals with psychiatric facility in the due course. Majority seek care for non-psychotic syndromes. Stigma is less intense, but people reject health workers (other than doctors) as healers. There is also poor follow up and lack of continuity in care from hospital to community.

Special programmes included the district-based suicide prevention programme in *Thrissur* and the *Ponnani* initiative which included a school mental health programme, old age care, suicide prevention and palliative care. Decentralisation of administration, political will of the local leadership and availability of a committed young psychiatrist in the community were the success formula behind the *Ponnani* initiative. There is an urgent need to include various aspects of mental health in medical education and to promote research in the area.

